# How *Klebsiella pneumoniae* controls its virulence

**DOI:** 10.1371/journal.ppat.1013499

**Published:** 2025-09-15

**Authors:** To Nguyen Thi Nguyen, Gareth Howells, Francesca L. Short

**Affiliations:** Department of Microbiology, Biomedicine Discovery Institute, Monash University, Clayton, Victoria, Australia; University of Pittsburgh, UNITED STATES OF AMERICA

## Abstract

The bacterial pathogen *Klebsiella pneumoniae* is a serious public health threat due to its propensity to develop antimicrobial resistance (AMR), the emergence of hypervirulent strains able to cause community-acquired infections, and the more recent development of convergent strains that exhibit both traits. Pathogenesis in *K. pneumoniae* is attributed to a range of largely horizontally-acquired virulence or fitness factors that collectively mediate immune evasion, attachment, intermicrobial competition and nutrition in different niches within the host. An outstanding research question is how expression of these factors is coordinated during infection, and how this regulatory control varies in genomically distinct lineages. Here we review recent progress in understanding the regulators and networks that control *K. pneumoniae* virulence or host fitness factor expression, discuss the role of plasmid–chromosome regulatory crosstalk in pathogenesis, and explore the potential of new global approaches to enhance our understanding. This knowledge will be instrumental in accurately predicting virulence from genome sequence in new emergent *K. pneumoniae* lineages, in order to track and manage this priority pathogen.

## Introduction

*Klebsiella pneumoniae* is a gram-negative Enterobacteriaceae family pathogen of global concern. Carbapenem and cephalosporin resistant *K. pneumoniae* have been classed as World Health Organization priority pathogens [1] and *K. pneumoniae* infections were responsible for an estimated loss of 31 million disability-adjusted life years in 2019 [2]. *Klebsiella pneumoniae* is characterized by extremely high genomic diversity and the ability to inhabit a broad range of host and environmental niches [3,4].

Despite its clinical spread, many common bacterial virulence factors such as toxins are rare or absent in *K. pneumoniae*, with its strategy for proliferation in the host instead described as “Going on the offense with a good defense” [5]. *K. pneumoniae* host colonization and pathogenesis are the subject of several recent reviews [6–10], and key features will be summarized here. *K. pneumoniae* possesses a suite of horizontally-acquired, ostensibly “plug and play” host fitness factors that contribute to infection by subverting host immunity, acquiring nutrients within the host, mediating intermicrobial competition, or a combination of these processes. These factors include a protective surface capsule, which can be hypermucoviscous, siderophores for iron acquisition, O-antigen, Type VI secretion systems and different types of fimbriae. Presence or absence of particular virulence factors, primarily carried on non-conjugative large virulence plasmids such as pLVPK [11], support the division of *K. pneumoniae* into hypervirulent (hvKp) and classical (cKp) (non-hypervirulent) isolates, where the former are those able to cause community-acquired disease (defined in research as an LD50 < 10^7^ in murine intranasal infection) [12,13]. Hypervirulence-associated factors include the siderophores aerobactin, salmochelin, and yersiniabactin, and the hypermucoidy-linked *rmpA* and *rmpA2* operons, which are associated with specific mobile genetic elements [14–16]. Recent genomics studies have been undertaken to characterize *K. pneumoniae* from a genomic epidemiology perspective, and to map the evolution and distribution of its pathogenicity determinants [17–19]. In general, our understanding of *K. pneumoniae* pathogenesis is hindered by the genomic diversity of the population. While approximately 2000–3000 genes are shared by 95% of isolates, more than half of *K. pneumoniae* genes vary between isolates [20]. This accessory genome includes approximately 30,000 protein coding genes and the majority of genes linked to virulence [10,20] and an outstanding area in understanding *K. pneumoniae* pathogenesis is how expression of its diverse set of virulence-associated genes is coordinated and controlled in different genetic backgrounds.

Current knowledge of *K. pneumoniae* pathogenesis is drawn from studies of well-established laboratory strains as well as more recent clinical isolates. Two widely used reference isolates for pathogenesis studies are strains Kp52.145 (a laboratory derivative of CIP52.145/B5055) [21] and ATCC43816 or its derivative KPPR1 [22], which have been used for foundational studies of capsule, hypermucoidy and iron acquisition, among others (e.g., [23–26]). Both are mouse-virulent, capsule type K2 isolates of clonal groups CG66 (Kp52.145) and CG493 (ATCC43816), respectively. Kp52.145 possesses two virulence-associated plasmids, is hypermucoid, and makes all four siderophores, while ATCC43816 lacks plasmids and does not produce aerobactin [21]. These isolates are from clonal groups only rarely encountered in the clinic, and since the 2000s many studies have explored pathogenesis in exemplars of the widespread hvKp lineage CG23, such as NTUH-K2044 [27], and SGH10 [28], both of which produce capsule of type K1.

The success of *K. pneumoniae* as a pathogen depends on its ability to fine-tune its behavior in different environments. For example, in a single human infection *K. pneumoniae* may spread from a mucosal surface such as the oropharynx, before colonizing the bloodstream and lungs [29–31] or from a patient’s own gastrointestinal microbiota to self-acquired infections such as UTIs [32,33]. In this process, populations of *K. pneumoniae* will experience multiple environments and selection pressures, including competition with other bacteria [34] and the host immune response [35], and diverse phenotypes will be more fit at different stages.

Complex signal transduction networks dictate the expression of the *K. pneumoniae* genes needed to thrive in different environments. Understanding these networks, and how they operate in different lineages of *K. pneumoniae*, could provide routes to reliably predict *K. pneumoniae* phenotype from genotype, or design new virulence-subverting therapeutics. In this review, we examine the main mechanisms that regulate expression of the primary *K. pneumoniae* virulence-associated factors, current knowledge of the role of accessory genes in controlling pathogenesis, and explore future research directions that would improve our understanding of this critical pathogen.

## Components of bacterial gene regulation networks

Bacteria use complex regulation hierarchies and networks to sense their environments and control gene transcription and translation in response. Gene expression is primarily controlled at the level of transcription initiation. At the most primary level, sigma factors (*σ*) dictate where RNA polymerase is able to initiate transcription by binding to specific promoter sequences [36,37]. A variety of *σ* factors bind to different conserved promoter sequences to control gene expression in response to different environmental stimuli [37]. Transcription factor proteins activate or repress transcription by binding to specific DNA recognition sequences, usually at or near a promoter, to alter interactions of RNA polymerase with that promoter [38,39]. Transcription factors are themselves activated or deactivated by either small molecule binding, or phosphorylation by histidine kinase partners as part of two-component systems (reviewed specifically for *K. pneumoniae* in [40]). The majority of transcription factors are local regulators that control one or a few genes, while some are global regulators with hundreds of genes under their direct control [38]. Precise and complex regulation circuits can be built from *σ* factors together with transcription factors, and many genes are regulated by multiple transcription factors that themselves respond to different cues [38,41].

Nucleoid-associated proteins such as H-NS and IHF provide another level of control over transcription by altering DNA accessibility, and are distinguished from transcription factors by their high abundance, relaxed DNA binding specificity and overall effects on DNA architecture and compaction [42]. Beyond transcription, small RNA (sRNA) regulation provides a global, fast-acting layer of control over which transcripts go on to be translated into proteins and exert their function [43]. Overall, bacteria use multiple sophisticated, intersecting regulatory systems to control which genes are expressed and when.

Despite the wealth of knowledge built over the past decades, understanding these regulatory networks is still challenging; in *Escherichia coli* MG1665 – a model bacterium for regulation – condition-specific gene expression can be predicted with up to 86% accuracy, but the specific direct regulators can be unambiguously linked for only 14% of transcription units [44].

Many regulators are highly conserved across different phyla, indicating central roles and deep evolutionary roots [45]. Regulator activities are typically extrapolated from well-studied organisms, particularly *E. coli* for which high quality curated resources are available [46]. However, the targets of specific regulators, and how they function within overall regulation networks, can vary substantially between species and even strains of the same species, as shown in both regulator-specific and global studies of various Enterobacteriaceae family bacteria [47–51]. An additional layer of regulatory complexity that is particularly relevant to *K. pneumoniae* is provided by mobile genetic elements; the accessory genome of this pathogen is crucial to both hypervirulence and multidrug resistance (MDR), and these elements can be tightly integrated into cellular regulation networks. Plasmid-encoded genes can be subject to complex regulation by core regulators, while accessory genome-encoded regulators are known to provide an important, infection-relevant layer of regulation in hvKp.

## Regulation of specific virulence factors

### Capsule

*K. pneumoniae* cells are enveloped in a coating of complex acidic polysaccharides which form the capsule [5]. *K. pneumoniae* produces Group I (*wzy*-dependent) capsules, and the biosynthesis of these capsules is determined by a chromosomal locus of three operons containing both conserved and variable genes [52,53]. Over 180 distinct capsule loci have been identified in *K. pneumoniae* [54,55]. Encapsulation is a strong determinant of *K. pneumoniae* virulence, with acapsular strains less virulent, and hypercapsular hvKp (hypermucoid and usually K1/K2 serotype) strains significantly more virulent [56]. The capsule primarily mediates virulence by protecting cells from opsonization and phagocytosis by the host immune system [5]. Capsule can also influence attachment and biofilm formation in *K. pneumoniae* by preventing attachment through masking of fimbriae [57], though some capsule types are apparently themselves involved in attachment [58]. hvKp strains are hypermucoid, and may also have capsule qualities that promote immune evasion, such as the absence of mannose residues in the K1 capsule type, thereby avoiding activation of the complement pathway by mannose binding lectins [59]. Note that while capsule is almost universally considered a host fitness factor, several intriguing studies have reported evolution of acapsular *K. pneumoniae* variants during infection, which can have increased fitness in specific host niches [60–63].

#### Transcriptional regulation by core genome-encoded global regulators.

**Fig 1:**
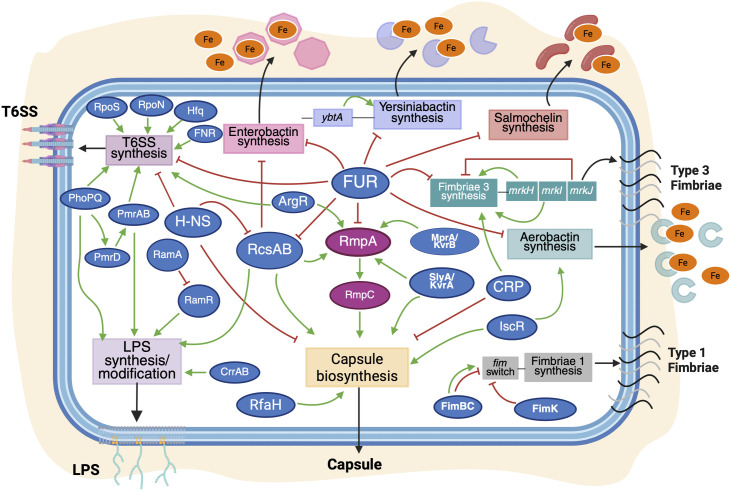
Overview of global and specific regulators controlling virulence factor expression in *K. pneumoniae.* Major virulence factor loci are indicated by boxes, and transcriptional regulatory proteins shown as blue ovals for core genome-encoded regulators, and purple ovals for accessory genome-encoded regulatory proteins. Green or red arrows indicate transcriptional activation or repression, respectively. A subset of global regulators including H-NS, the Rcs system, and Fur are involved in regulation of multiple factors. Created in Biorender.

Capsule is constitutively expressed, but can be upregulated or repressed in response to various environmental cues. Capsule synthesis is controlled at the level of transcription initiation by a network of chromosome-encoded global regulators [64,65]. The Rcs phosphorelay system is a membrane stress–response system conserved across the *Enterobacteriaceae*, which regulates many genes, including colanic acid in *E. coli* and capsule production in *K. pneumoniae* [66]. RcsAB positively regulates capsule expression [67,68] and binds to the promoters of the capsular polysaccharide biosynthesis genes *galF* and *manC* (Table 1 and Fig 1) [69]. Capsule production is also regulated in response to glucose levels; when cyclic AMP levels are high (indicating low exogenous glucose), capsule production decreases through the action of catabolite repressor protein (CRP), which binds directly to promoters within the capsule locus and also regulates expression of *rcsA* [70]. Iron levels influence capsule expression through repression of *rcsA* transcription by the iron-dependent regulator, Fur, and the small RNA, RyhB [71,72], while an additional iron-responsive transcriptional activator, IscR, promotes capsule production by directly binding to capsule locus promoters (Fig 1) [73].

**Table 1:** Summary of regulators referenced in the manuscript.

Regulator	Description	Kp strain	Reference
***Capsule and hypermucoviscosity***			
RcsAB	Positive regulator of capsular polysaccharide biosynthesis, *galF* and *manC* promoters	NTUH-K2044; 889/50 (O1:K20); KPPR1S	[67–69,79]
Fur	Negative regulator of *rcsA* in iron rich conditions, negative regulator of *rmpA*	CG43 (clinical isolate of K2 serotype)	[72]
IscR	Positive regulator of cps locus expression	CG43S3	[73]
CRP	Binds to promoters within capsule locus and regulates *rcsA* expression, negative regulator of capsule expression	CG43S3; NTUH-K2044	[70,94]
H-NS	Represses capsule gene transcription	Kpn 123/01 (serotype K39); NTUH-K2044	[64,74]
KvrA/SlyA	Positive regulator of capsule gene expression through RmpA	KPPR1S; NTUH-K2044; classical Kp strain MKP103	[64,76,79]
KvrB	Positive regulator of capsule gene expression through RmpA	KPPR1S; classical Kp strain MKP103	[76,79]
RmpA	Reported positive regulator of CPS production, requires RcsB for expression	CG43; NTUH-K2044; KPPR1S	[23,77,79,95]
RmpA2	Positive regulator of CPS production	CG43	[78,96]
RmpC	Promotes capsule expression, encoded downstream of *rmpA*	KPPR1S	[53,79]
ArgR	Positive regulator of hypermucoidy through control of *rmpADC* expression	KPPR1S	[86]
OmpR/EnvZ	Two component system, deletion reduces hypermucoidy	NTUH-K2044; ATCC43816	[64,88]
ArcAB	Two component system, deletion reduces capsule production	NTUH-K2044; ATCC43816	[64]
KbvR	Positive regulator of capsule biosynthesis	NTUH-K2044	[89]
KvhA/KvhR	Positive regulator of capsule biosynthesis	CG43S3	[90]
RfaH	Positive regulator of capsule expression through interactions with RNA polymerase, prevents early termination of capsule transcripts	TOP52	[91]
OmrB	sRNA, negative regulator of capsule biosynthesis. Base-pairs to the transcript of *kvrA/slyA*	SGH10	[93]
ArcZ	sRNA, negative regulator of hypermucoviscosity. Binds to transcripts of *mlaA* and *fbp*	ATCC43816	[92]
RyhB	Small RNA, positive regulator of capsule (via activating orf1 and orf6 of K2)	CG43S3 (streptomycin resistant CG43)	[71]
***Lipopolysaccharide synthesis and modification***			
PhoP/Q	Two component system, regulates Lipid A modification genes	CG43; clinical colistin resistant isolates	[40,97,98]
RcsA/B	Two component system	NTUH-K2044; CG43	[99]
RfaH	Enhances transcriptional elongation of *waaQ* and *rfb* operons	NTUH-K2044	[91]
PmrA/B	Two component system	Clinical isolates (KKBO-1 and KKBO-4)	[100]
CrrA/B	Two component system	Clinical colistin-resistant isolates	[101]
RamA	Activator of *lpxC*, *lpxL*-2, and *lpxO*	Ecl8	[102]
***Siderophores***			
Fur	Global regulator of siderophore synthesis, represses siderophore production in iron rich conditions	CG43; SGH10; NTUH-K2044	[72,87,103,104]
RcsAB	Two component system, represses expression of *entC*	NTUH-K2044	[104]
RyhB	sRNA, can promote expression of aerobactin and enterobactin	CG43S3	[71]
IscR	Transcription factor, can promote aerobactin production in response to low iron	CG43	[73]
***Fimbriae***			
FimK	Binds inverted repeats flanking *fimA*, affects phase variation. Direction of regulation may be context-dependent as the two studies suggest opposite effects.	TOP52 (cystitis isolate); CG43S3	[105,106]
MrkH	Local activator of type 3 fimbriae *mrk* cluster, c-di-GMP dependent	AJ218 (K54 clinical isolate)	[107]
Fur	Promotes *mrkH* expression in response to low iron	CG43	[108]
IscR	Repressor of *mrkH* expression	CG43S3	[109]
IroP	Virulence plasmid encoded suppressor of type 3 fimbriae expression	SGH10; tested in some other hvkp strains	[87]
H-NS	Represses *mrkA* transcription	Kpn 123/01 (serotype K39)	[74]
KfpR	Transcriptional repressor of *kpf* fimbriae expression	UKP8 (UTI isolate)	[110]
***Type 6 secretion***			
PhoPQ	Two component system, Positive regulator of T6SS	CIP52.145 (hereafter Kp52145)	[111]
PmrAB	Two component system, Positive regulator of T6SS	CIP52.145	[111]
Hfq	Positive regulator of T6SS	CIP52.145	[111]
Fur	Negative regulator of T6SS in low iron	CIP52.145; KPPR1S	[111,112]
RpoS	Sigma factor, Positive regulator of T6SS	CIP52.145	[111]
RpoN	Sigma factor, Positive regulator of T6SS	CIP52.145	[111]
H-NS	Negative regulator of T6SS	CIP52.145	[111]
MgrB	Negative regulator of T6SS	CIP52.145	[111]
RcsB	Negative regulator of T6SS	CIP52.145	[111]
ArgR	Positive regulator of T6SS in response to arginine	KPPR1S	[112]
FNR	Positive regulator of T6SS in response to low oxygen	KPPR1S	[112]

Regulators referenced in the manuscript, including identifiers, descriptions, strains in which experimentation was done and cited literature.

The abundant nucleoid-associated protein H-NS represses capsule production [74], and its activity is thought to be counteracted by the regulator SlyA (called KvrA in *K. pneumoniae*) at high temperatures based on work in *E. coli* [75]. SlyA/KvrA is known to regulate capsule in both hvKp and cKp, though its mechanism of action has not been characterized in depth [64,76].

#### Accessory genes and regulation of hypermucoidy.

**Fig 2:**
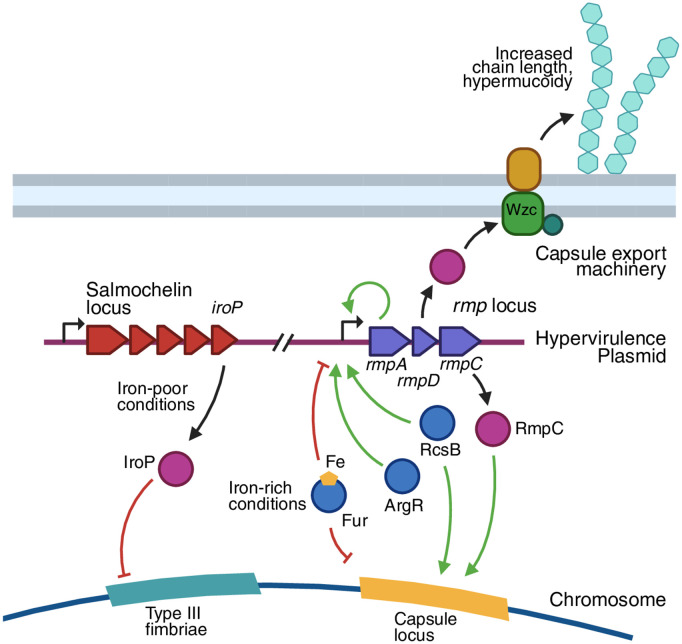
Plasmid–chromosome cross talk in regulating virulence factor expression in hypervirulent *K. pneumoniae.* The canonical hypervirulence plasmid contains both the Salmochelin and *rmp* loci. IroP is expressed from downstream of the salmochelin synthesis genes, and suppresses expression of the chromosomal Type 3 fimbrial genes when iron levels are low. The *rmp* locus encodes three proteins that collectively act to increase capsule transcription and promote a change in capsule structure. Both *rmp* and the capsule loci are repressed by Fur in high iron conditions, and are regulated by the Rcs system. In addition, the *rmpA* promoter is activated by the core genome-encoded regulator ArgR. Created in Biorender.

Capsule production is also subject to regulation by components of the *K. pneumoniae* accessory genome, and this was recognized as early as the 1980s with the identification of RmpA (for Regulator of mucoid phenotype) as a plasmid-encoded mucoidy regulator [23]. RmpA and RmpA2 are homologous regulators usually encoded on hypervirulence plasmids of *K. pneumoniae* [16], though in many strains one of the genes is truncated [77,78]. The presence of RmpA was shown to increase capsule production in early functional studies, which also revealed that this increase required a functional RcsAB system [77]. RmpA and RmpA2 promote the hypermucoid phenotype that marks hvKp. Recent work in KPPR1S has revealed the details of how hypermucoidy and capsule production are connected. The *rmpA* gene is encoded in an autoregulated operon with two additional genes, *rmpD* and *rmpC*, encoding a small protein and a transcriptional regulator, respectively [53,79]. RcsB is required for full expression of this operon [77,79]. RmpC promotes transcription of capsule genes [Fig 2], and is likely responsible for the increase in capsule expression previously attributed to RmpA [79]. The RmpD protein does not affect capsule gene transcription, but promotes synthesis of longer and more uniform capsule polysaccharide chains through direct interaction with the capsule export machinery [53,80]. This change in capsule architecture underpins the hypermucoid phenotype [80], and similar changes in capsule structure leading to hypermucoidy have also been observed in *K. pneumoniae* strains carrying certain spontaneous mutations in the capsule export protein, Wzc [81–83]. RmpA2 has been studied in the context of the hypervirulence plasmids pK2044 and pLVPK, where it is encoded in an operon with a *rmpD* homologue (RmpD2) and a truncated *rmpC* gene. Like *rmpADC*, this locus was shown to increase hypermucoidy and capsule production even though it does not encode a functional copy of RmpC [84], and an additional study confirmed the function of multiple *rmpA* locus variants in KPPR1S [16]. The effect of both *rmp* loci can vary depending on strain background and capsule type [84]. Though the underlying reasons for strain specificity are not completely understood, *rmpA* promoter mutations that abrogate hypermucoviscosity have been observed in some hypervirulent carbapenem-resistant *K. pneumoniae* (hv-CRKP) strains [85].

The discovery of the molecular basis for the hypermucoid phenotype opened the way for studies specifically addressing regulation of this phenomenon, and the conserved regulator ArgR was recently identified as an arginine-dependent activator of hypermucoidy that works through promoting expression of *rmpADC* [86]. In another example of tight regulatory integration between core and accessory genome components, both RmpA and RmpA2 are also controlled by the global regulator Fur [72,87].

#### Further transcriptional regulation.

Additional regulators of capsule or hypermucoidy have been identified through high-throughput screens [64,65] and targeted genetic studies, including the global two-component regulatory systems ArcAB (linked to aerobic/anerobic transitions) and OmpR-EnvZ (osmotic stress response) [88], and presumed specific regulators KvrB [76,79], KbvR [89], and KvgSA [90] (Table 1). These studies highlight how tightly tuned capsule production is to the environment, as well as the various metabolic and structural components needed for maximum capsule production. Note that the regulators mentioned in this section can increase or decrease capsule but are not known to suppress capsule completely. In long-term host infections, particularly UTIs, acapsular isolates arise through mutation relatively frequently and appear to have a fitness advantage [60–62], illustrating how relevant phenotypes *in vivo* can be achieved through mutation as well as genetic regulation.

#### Post-transcriptional regulation.

Capsule production is also regulated after transcription initiation. For instance, the transcription elongation factor, RfaH, is required for efficient capsule gene expression [64,91]. Global RNA interaction profiling in hvKp has revealed capsule and hypermucoidy as hubs for post-transcriptional regulation by small RNAs; one study identified 18 different sRNAs that influence mucoviscosity when overexpressed [92], while another study showed the same for 5 of 8 tested sRNAs [93]. Two newly-characterized, capsule-regulating sRNAs of *K. pneumoniae*, OmrB and ArcZ, have been investigated in depth and their molecular targets identified. OmrB suppresses both capsule production and hypermucoidy, and base-pairs to the transcript of the KvrA/SlyA regulator (Fig 1) [93]. ArcZ suppresses hypermucoidy when overexpressed by targeting transcripts of the phospholipid trafficking protein, MlaA, and the gluconeogenesis enzyme, Fbp [92]. Note that MlaA was previously linked to hypermucoidy through high-throughput genetic screening [64]. ArcZ expression is itself regulated by carbon source availability through CRP [92]. Notably, these global studies of *K. pneumoniae* sRNA regulation identified both novel potential species-specific sRNAs, as well as new regulatory circuits involving conserved RNAs, which highlights the need for direct studies of pathogens of interest, even when regulators are well-characterized in other species.

Though by far the best-studied of the *K. pneumoniae* virulence factors, there are important limitations to our current understanding of capsule regulation. The majority of studies do not differentiate between regulation of hypermucoidy and regulation of capsule gene expression, with this distinction being recognized relatively recently [79]. Direct comparisons between studies are hindered by the variety of growth conditions and media used, and shared regulators can have strain-specific impacts [64,65,84]. Further work will be needed to understand which regulatory circuits hold across the entire *K. pneumoniae* species or are specific to hvKp or *rmpA*-containing strains, and how their activity plays out in varied and changing environmental conditions.

### Lipopolysaccharide

As a gram-negative bacterium, *K. pneumoniae* possesses an asymmetric outer membrane with the outermost face containing lipopolysaccharides (LPS), which are comprised of a hydrophobic membrane-anchored lipid A component (encoded by the *lpx* gene cluster), a core oligosaccharide component (encoded by the *waa* cluster), and a terminal, strain-variable O-antigen (encoded by the *wb*/*rfb* cluster) [113]. LPS is strongly immunogenic, and *K. pneumoniae* can evade the immune response through covalent modifications of the lipid A moiety [114,115], or variations of the O-antigen [25,116]. Like capsule, LPS is constitutively expressed, but is also specifically regulated – or modified – in order to remodel the cell envelope for immune evasion or environmental survival. Very few regulators of LPS transcription have been specifically confirmed in *K. pneumoniae*. Fur binds to the promoter of the core LPS gene *uge* to repress transcription in iron-rich conditions [117], while the antiterminator RfaH is required for full transcription of the O-antigen genes [91]. Note that in *E. coli*, LPS and O-antigen expression is controlled by many regulators [118], so it seems likely that additional pathways active in *K. pneumoniae* will be uncovered with more research.

Regulation of lipid A modification has been studied in more depth*.* Several two-component systems (PhoP/PhoQ, PmrA/PmrB, CrrA/CrrB) have been shown to have direct or indirect involvement in LPS modifications that lead to resistance to colistin [98–100]. The Rcs system has also been described as mediating these colistin-induced transcriptional changes together with PhoPQ, where the Rcs system downregulates *phoP* transcription and the PhoPQ system promotes the expression of *rcsD* and *rcsC* (and presumably the operonic *rcsB*) [119]. An additional important regulator identified in *K. pneumoniae* is RamA, which can bind to and activate the promoters of the *lpxC, lpxL-2*, and *lpxO* genes – all of which encode proteins involved in Lipid A modifications [102]. RamA-overexpressing mutants have increased dissemination through the host [102].

### Siderophores

In the host environment, iron is limited. Therefore, microbes compete for iron by producing siderophores that bind Fe^3+^ [120]. *K. pneumoniae* can produce up to four different siderophores – enterobactin, yersiniabactin, aerobactin and salmochelin [5]. Enterobactin is encoded on the core *K. pneumoniae* genome [19,21] on two operons comprising biosynthetic and transport genes [120]. It has very high iron affinity but is sequestered by host lipocalin-2 during infection [121]. Therefore, production of additional accessory genome-encoded siderophores that are not recognized by lipocalin-2 increases virulence, in a manner that strongly depends on host niche [10,26,122–127]. Additional siderophores include salmochelin, which is a glycosylated derivative of enterobactin [128], and yersiniabactin and aerobactin which are the chemically distinct phenolate and hydroxamate siderophore types, respectively [120]. Yersiniabactin is usually present on a virulence-associated integrative conjugative element, ICEKp, and is present in 90% of hvKp and approximately 45% of cKp [14,129,130]. Aerobactin and salmochelin are usually co-localized on the hypervirulence plasmid, and are present in 90% of hvKp but rare in cKp [14,130]. The loci for all four siderophores include more than one transcriptional unit. Unlike capsule and LPS, siderophores are not constitutively expressed [72].

Siderophore synthesis is controlled by both global and local regulators (Table 1). All four *K. pneumoniae* siderophore genes are repressed by Fur under conditions of high iron [72,87,103]. Enterobactin production is additionally regulated by the RcsAB two-component system, which has been shown to directly target the promoter of the first biosynthetic gene of the locus, *entC* [104]. Fur also represses *rcsA* transcription, thereby regulating enterobactin gene transcription directly and indirectly. The Fur-regulated small RNA, RyhB, participates in activation of some Fur-regulated gene transcripts, as well as capsule transcripts [71,87]. In addition, the Fe-S cluster-containing transcription factor, IscR, has been reported to regulate aerobactin production in its *apo* form [73].

Siderophore production has been shown to be controlled by additional regulatory systems in other *Enterobacteriaceae* species, such as the yersiniabactin-specific feedforward regulator YbtA [131], the anaerobiosis/redox-sensitive regulatory system ArcAB [132], and the osmolarity-sensing system OmpR-EnvZ [133]. While it is likely that some of these regulators also control siderophore expression in *K. pneumoniae*, this has not yet been tested. Overall, there is a lack of systematic information, as current studies have been performed in various *K. pneumoniae* strains with different siderophore complements. Regulation differences may contribute to the variation in the virulence contributions of individual siderophores across strain backgrounds, or the dominance of aerobactin in the secreted siderophore pool of at least one hvKp strain [124,125]. Another interesting feature of siderophore regulation is the degree of involvement of regulators also known to target capsule production, such as Fur, IcsR, and RcsAB (Fig 1).

### Fimbriae

Fimbriae (pili) are thin polymers on bacterial surfaces that mediate adhesion to biotic and abiotic surfaces [134], and contribute to both pathogenesis and environmental persistence through the initiation of biofilm formation [134–136]. In *K. pneumoniae*, most strains can express both type 1 and type 3 fimbriae [137]. Type 1 fimbriae are hair-like fibers (width approximately 7 nm, length 0.2–2 µm [138]) essential for pathogenesis in *K. pneumoniae* urinary tract infections [137], and are encoded by the *fim* cluster (structured *fimBEAICDFGHK* in *K. pneumoniae*). These adhesins are composed of FimA polymers capped with the mannose binding FimH [136]. Type 1 fimbriae expression is phase variable and controlled by the inversion of the *fimA* promoter region [105,136]. This region is termed the *fim* switch – in *E. coli*, inversion is controlled by the FimB and FimE recombinases [136,138] and this activity is presumed to be the same in *K. pneumoniae*. *K. pneumoniae* contains an additional gene, *fimK* [136], which is not found in other Enterobacteriaceae, and encodes a protein that promotes inversion of the *fim* switch to the OFF position [105], and has also been reported to directly bind to the promoter of *fimA* to promote its transcription [106]. In *E. coli* the *fim* switch is also regulated by the global regulators H-NS and Fis [139,140].

Type 3 fimbriae are thinner (4–5 nm width) than type 1 fimbriae, and are important for adhesion and biofilm initiation [137]. Type 3 fimbriae are encoded by the *mrk* cluster, where within the cluster, *mrkA* encodes the fimbrial subunit and *mrkD* encodes the adhesin. Expression of the *mrkABCDF* cluster is regulated by the products of the downstream genes, *mrkHIJ*, and both operons are subject to control by global regulators linked to nutrient levels. The *mrkH* gene encodes an activator that requires the biofilm-related second messenger cyclic-di-GMP for activity [107]; c-di-GMP levels are themselves related to carbon availability via the cAMP-CRP system [141]. Type 3 fimbriae production is also regulated in response to iron, where expression of *mrkH* is activated by Fur [108] and repressed by IscR [109]. In addition to the aforementioned nutrient-responsive regulators, the nucleoid protein H-NS has also been shown to control Type 3 fimbriae by repressing *mrkA* transcription [74]. In hvKp, Type 3 fimbriae synthesis is also regulated through plasmid–chromosome cross talk involving the protein IroP [87]. This small protein is encoded within the salmochelin biosynthesis operon and is therefore induced in iron-limited conditions. IroP suppresses expression of Type 3 fimbriae, thereby strengthening the inverse regulation of Type 3 fimbriae and hypermucoid capsule production in response to iron availability. This regulatory circuit is conserved in some, but not all hvKp strains [87].

In addition to the *fim* and *mrk* clusters, there are a range of fimbriae gene clusters in *K. pneumoniae* that are less well characterized [142,143] including the *kpf* cluster, which encodes a less common type 1-like fimbriae [144] together with its own transcriptional repressor gene *kpfR* [110].

### Colibactin

Colibactin is a genotoxin produced by some *K. pneumoniae* isolates as well as other members of the Enterobacteriaceae, that cross-links host DNA and induces double stranded DNA breaks [145]. The presence of colibactin-encoding *K. pneumoniae* has been suggested as a colorectal cancer biomarker [145]. Colibactin is encoded on an integrative conjugative element – ICE*Kp10* [14] – that is strongly associated with hvKp clonal group 23 [28], and can contribute to *K. pneumoniae* hypervirulence [146]. Regulation of colibactin production in *K. pneumoniae* has not been studied specifically. In *E. coli*, colibactin expression is controlled by a local transcriptional regulator, ClbR [147], and is also regulated in response to iron availability through the activity of Fur and RhyB [148,149]. The ClbR regulator is also present in *K. pneumoniae* [145].

### Type VI secretion system

The type VI secretion system (T6SS) is a bacterial nanomachine that translocates effector substrates into host cells [150]. In *K. pneumoniae*, T6SS contributes to attachment to host cells and plays a role in competition with other microbes, particularly in the highly competitive environment of the gut [112,151,152]. There is significant variability in T6SS amongst *K. pneumoniae* strains, with strains carrying between 0 and 4 T6SS gene clusters, as well as ‘orphan’ T6SS genes that lie outside these clusters – this underscores the importance of coordinated regulation of multiple loci [150,153]. T6SS are associated with hvKp [154,155] and have been investigated as a biomarker for progression from colonization to bloodstream infection [155]. Regulation of *K. pneumoniae* T6SS has been studied in Kp52145 [111] and KPPR1 [112]. The first study investigated one of the T6SS loci present in Kp52145 in detail and found various environmental conditions that promoted its expression including low oxygen, high ionic strength, iron starvation and 37 °C temperature; the two other T6SS of this strain did not share all of these transcriptional responses. T6SS expression was positively regulated by the RNA chaperone Hfq, the two-component systems PhoPQ and PmrAB, and the RpoS and RpoN sigma factors, and negatively regulated by H-NS, MgrB, Fur, and RcsB (Table 1). This study did not determine which regulators acted directly or indirectly, PhoPQ was demonstrated to act downstream of MgrB and RcsB and to activate T6SS expression in response to antimicrobial peptides [111]. A second study, of KPPR1, investigated the regulators ArgR, FNR, and Fur [112]. Each of these regulators was shown to control T6SS transcription in response to environmental signals relevant to the gut; ArgR mediated induction by arginine, FNR was required for induction in response to low oxygen, and Fur mediated T6SS induction in low-iron conditions, and in each case direct transcription factor-promoter binding was shown [112]. Presumably, the response to several individual cues ensures robust upregulation of T6SS transcription in the gastrointestinal tract.

## Plasmid–chromosome cross talk in *K. pneumoniae* regulation

*K. pneumoniae* is notable for its acquisition and carriage of plasmids, and these elements can be strongly embedded into cellular regulatory networks. Some *K. pneumoniae* plasmids, particularly the hypervirulence plasmids [11,19], have a very long history of association in specific lineages, while others are recently acquired and have had less time for host adaptation [156]. Plasmids can influence cellular regulation by providing regulators directly, or through general downstream effects of their encoded gene functions or the metabolic cost of their carriage. The impact of plasmids on regulation is best illustrated by hvKp. Various horizontally-acquired factors, including the regulators RmpA and RmpA2, siderophores aerobactin, yersiniabactin and salmochelin, and metabolic transporter *peg-344*, contribute to the hvKp pathotype to different extents, with RmpA, followed by aerobactin, having the strongest individual effect [124]. With the exception of yersiniabactin, all of these hypervirulence-associated factors are encoded on the canonical hypervirulence plasmid, pLVPK (Fig 2) [11], though note that RmpA/RmpA2 can also be located on integrative conjugative elements [16].

Plasmid pLVPK is highly integrated into the regulatory network of major hvKp lineages through the plasmid-encoded regulators discussed above with reference to capsule and fimbriae; RmpA controls both capsule biosynthesis (via the chromosomal capsule biosynthesis genes) and hypermucoidy (through the operonic *rmpD* gene) [53,157], while IroP suppresses transcription of Type 3 fimbriae genes in iron-poor conditions [87] (Fig 2). These regulators provide a striking example of how mobile genetic elements can confer clinically important phenotypes through regulation, rather than provision of virulence factors *per se*.

Questions of chromosome–plasmid cross talk are particularly relevant to convergent *Klebsiella pneumoniae* – those strains possessing genes for both hypervirulence and resistance to last-line antibiotics. Convergent *K. pneumoniae* strains can be hypervirulent lineages that have acquired drug resistance [158], or drug-resistant lineages with acquired virulence genes [159], and have been widely identified. Many convergent isolates have lower virulence than expected given their complement of virulence factors [160–163], suggesting there is some important lineage-specific activity or regulation that limits the amount or effect of these factors during infection. A few studies have examined this phenomenon in detail in specific strains and lineages.

In a large collection of *K. pneumoniae* ST11 (an MDR lineage), many isolates contained plasmids derived from the hypervirulence plasmid pK2044, though the virulence of these isolates was variable [162]. Further investigations found that the plasmid had a deletion of three complete genes of unknown function (named *hp2*, *hp3*, and *hp4*), and this deletion resulted in increased oxidative stress resistance through upregulation of several chromosomal genes, which in turn led to increased proliferation in macrophages [164]. hvKp strains are typically poorly internalized by macrophages due to their capsule size, so this finding may reflect an adaptation of an hvKp plasmid to suit the pathogenesis mechanisms of a different lineage.

The effects of some MDR plasmids have also been assessed. One study showed that in a hvKp strain, laboratory acquisition of a carbapenemase containing MDR plasmid resulted in reduced capsule, hypermucoidy and serum resistance *in vitro*, and elicited large transcriptional changes that may reflect a fitness cost [165]. Another found that acquisition of large AMR plasmids in two cKp representatives resulted in transcriptional changes to counter fitness costs, rather than mutations [166]. Finally, the epistatic interactions of the widespread carbapenem resistance plasmid, pOXA-48, have been examined in depth across various Enterobacteriaceae including *K. pneumoniae* [167–169]. Carriage of this plasmid is usually fitness-neutral [168], and interestingly, transcriptional changes associated with plasmid carriage are variable and not directly associated with fitness cost [169]. In *K. pneumoniae* specifically, a pOXA-48-encoded regulator was shown to induce expression of a small chromosomal operon and result in increased fitness, though the precise role of the regulated operon during infection is not known [169].

Overall, studies so far demonstrate that plasmid–chromosome regulatory interactions are frequent and have important effects, but are very difficult to predict even with well-characterized plasmids. This highlights the need for studies of regulatory networks in a diverse collection of strains, as well as functional assessments of plasmid encoded transcription regulators.

## New methods for untangling regulatory networks

Single gene molecular approaches, usually examining the effect of genetic deletion of a regulator-encoding gene combined with experiments showing direct regulator-target interactions, are the gold standard for understanding regulation. Genome-scale approaches to examine gene expression (RNAseq) and transcription factor binding (chromatin immunoprecipitation coupled sequencing or ChIP-seq) have been applied effectively to understand the targets of specific regulators, and advanced transcriptomics methods also offer the opportunity to profile operon structure at high resolution [170], including unusual configurations such as non-contiguous operons [171]. These approaches cannot scale sufficiently to reveal how regulatory networks operate across the phylogenetic diversity of *K. pneumoniae*. In general, approaches to define the activity and targets of a specific regulator are more developed than “target first” approaches to agnostically identify what controls a gene of interest. However, there have been promising advances in regulator screening methods that may accelerate this discovery in future. Here we will briefly discuss advances in this area with reference to methods aimed at identifying transcriptional regulators.

Transposon insertion sequencing is a well-established method for massively parallel mutagenesis and fitness assessment of bacteria, and has been used to identify regulators by selecting based on a phenotypic readout rather than growth. This technique has been applied to *K. pneumoniae* capsule [64] as well as phenotypes such as motility or dye efflux in other bacteria [172]. Transposon directed insertion-site sequencing (TraDIS) can be expanded to define regulators of phenotypes that cannot be directly selected by using transcriptional reporter activity, while an approach known as SorTnSeq was originally applied to *Serratia* [173]. CRISPR knockdown-based screening methods operate on similar principles but offer advantages in the ability to examine essential gene activities and to assay multiple reporters simultaneously. In one elegant design deployed in *E. coli* named PPTP-seq (pooled promoter responses to TF perturbation sequencing), CRISPRi guided RNAs to knock down various transcription factors were paired with transcriptional reporter fusions on the same plasmid, allowing regulator suppression to be linked to expression changes for a library of promoters at a global scale [174]. This method was used to identify new regulatory circuits in *E. coli* and recapitulated the bulk of known interactions. A limitation of SorTnSeq, PPTP-Seq and related methods is that they cannot show if a regulator effect is elicited by direct or indirect promoter binding. Other methods incorporate cross-linking and mass spectrometry (with or without parallel reporter assays) to assign transcription factor-promoter interactions *en masse* [175,176]. Finally, building on dramatic advances in protein fold prediction, *in silico* methods to predict protein–protein or protein–DNA binding have been developed (e.g., [177–179]) and may prove highly valuable for accelerating or streamlining identification of regulator interactions in *K. pneumoniae*, and predicting how these operate in diverse strains.

In addition to methods aimed at defining regulatory proteins, there have also been dramatic advances in global methods to identify small regulatory RNAs and their targets, such as the RIL-seq and CLASH (both based on sequencing of ligated sRNA-target molecules) techniques used to identify the capsule or hypermucoidy-regulating sRNAs described above [92,93]. An additional RIL-seq study found a *K. pneumoniae*-specific small RNA regulator of cell division [180], further highlighting that extrapolation from other *Enterobacteriaceae* is not sufficient to understand behavior control in this pathogen.

The availability of genuinely high-throughput methods to profile regulation networks in terms of both expression and transcription factor binding is an exciting development likely to reveal new aspects of *K. pneumoniae* biology in coming years. However, challenges remain in applying these methods. Functional genomic approaches are rarely one-size-fits-all so it is difficult for researchers to integrate and interpret findings across studies, with significant further experimentation needed to define which interactions are biologically important.

## Concluding remarks

*K. pneumoniae* is a complex bacterium notable for its genomic and phenotypic flexibility. These properties underscore the devastating impact of *K. pneumoniae* as a pathogen. As our understanding of *K. pneumoniae* genomics, epidemiology and molecular pathogenesis comes of age, our understanding of regulation has also increased. Regulation of virulence factors in *K. pneumoniae* is now known to involve multiple conserved regulators working in concert with horizontally-acquired factors that can dramatically influence pathogenesis. Regulators with conserved activity can have strain-specific impacts, as illustrated by the unpredictable virulence of convergent *K. pneumoniae* strains. Analyzing the similarities and differences in the regulation of virulence factors can provide hints as to their roles and interactions during infection, as illustrated by the reciprocal regulation of capsule and fimbriae production. Untangling the regulatory networks that control *K. pneumoniae* pathogenesis, as well as how these operate across diverse strain backgrounds, represents a major research challenge that will greatly help with efforts to predict *K. pneumoniae* phenotype from genotype in future.

## Abbreviations

AMR: antimicrobial resistance
CRP: catabolite repressor protein
MDR: multidrug resistance
sRNA: small RNA
TraDIS: transposon directed insertion-site sequencing
